# Synthetic biology strategies for microbial biosynthesis of plant natural products

**DOI:** 10.1038/s41467-019-09848-w

**Published:** 2019-05-13

**Authors:** Aaron Cravens, James Payne, Christina D. Smolke

**Affiliations:** 1 Department of Bioengineering, Stanford University, 443 Via Ortega, MC 4245, Stanford, CA 94305 USA; 2 Chan Zuckerberg Biohub, 499 Illinois St, San Francisco, CA 94158 USA

**Keywords:** Metabolic engineering, Metabolic engineering

## Abstract

Metabolic engineers endeavor to create a bio-based manufacturing industry using microbes to produce fuels, chemicals, and medicines. Plant natural products (PNPs) are historically challenging to produce and are ubiquitous in medicines, flavors, and fragrances. Engineering PNP pathways into new hosts requires finding or modifying a suitable host to accommodate the pathway, planning and implementing a biosynthetic route to the compound, and discovering or engineering enzymes for missing steps. In this review, we describe recent developments in metabolic engineering at the level of host, pathway, and enzyme, and discuss how the field is approaching ever more complex biosynthetic opportunities.

## Abstract (web summary)

Engineering microbial cell factories for the production of useful plant natural products (PNPs) is a resource-conserving and environmentally-friendly synthesis route. Here, the authors review recent developments that enable engineering of hosts, pathways, and enzymes to make PNPs and PNP derivatives.

## Introduction

The field of metabolic engineering endeavors to create a green manufacturing industry based on bioproduction of commodity chemicals in cell factories. Plant natural products (PNPs) are especially important targets because of their utility as flavors, fragrances, and medicines, but can be challenging to synthesize due to stereochemical complexity. PNPs are produced via specialized plant metabolism involving numerous enzymes from diverse classes that enzymatically transform central metabolites into secondary metabolite compounds such as the analgesic morphine and antimalarial artemisinin. Many PNPs are obtained from processed plant biomass, requiring substantial land, water, and time investment, and which introduces insecurity in supply chains due to variability in crop yields resulting from pests or extreme weather. Furthermore, intermediates in a PNP biosynthetic pathway are often unavailable from the host plant, thus complicating efforts to produce novel derivatives of the PNP of interest. Microbial production of PNPs can overcome these challenges by enabling (1) on-demand production capabilities associated with microbial cells, (2) scalable and controlled production in fermentation facilities, and (3) the capacity to produce PNPs and PNP intermediates at higher purity or yield than those provided by the native plant host. In addition, microbial production of PNPs can serve as a discovery platform to synthesize novel derivatives of PNPs and gain insight into enzymes involved in plant secondary metabolism.

**Fig. 1:**
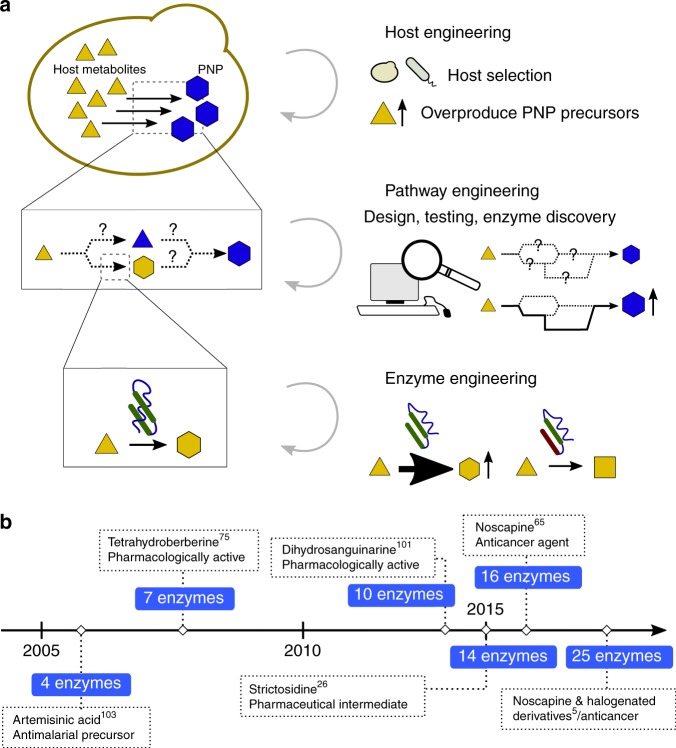
Metabolic engineering at multiple levels has enabled engineering of increasingly complex heterologous PNP pathways. **a** Heterologous production of a PNP in a microbial host can involve engineering at three different scales: host, pathway, and enzyme. **b** Timeline of examples of notable yeast-produced PNPs, showing increasing pathway length accomplished over time. Labels show product name or compound utility

Metabolic engineering to produce a particular PNP relies on iterative engineering cycles of design, build, and test referred to as the DBT cycle^1^. At the level of the host, DBT includes selection and engineering of the host to overproduce PNP-precursor metabolites in sufficient quantity; at the pathway level, a biosynthetic route to produce the PNP is determined and candidate enzymes are tested or discovered; and at the enzyme level, protein engineering may be warranted to improve function or produce derivative compounds (Fig. 1a).

Advances in synthetic biology and enabling technologies like DNA synthesis^2^, sequencing^3^, and analytical techniques^4^ have accelerated the DBT cycles for metabolic and protein engineering to the point where both can be deployed to engineer the biosynthesis of a particular molecule. Indeed, the complexity of PNP pathways being discovered and engineered has steadily increased over the past 20 years (Fig. 1b), highlighted by a recent example of a 25-enzyme pathway for the anticancer compound noscapine reported in 2018^5^. Once a strain has been developed that produces a small amount of the desired product, strategies to engineer that strain for industrial-scale titers of the product can then be employed; these strategies have been reviewed elsewhere^6^, and the focus of this review will be on initial engineering strategies producing at least detectable concentrations of the desired PNP and/or novel PNP derivative which verify pathway viability. Heterologous PNP biosynthesis and application of DBT require judicious selection and engineering of the production host, the biosynthetic pathway, and the individual enzymes composing the pathway. In this review we discuss recent examples and technologies that enable engineering of hosts, pathways, and enzymes to make PNPs and novel PNP derivatives and the technological advances on the horizon that are expected to further accelerate this field. In the coming years, we expect researchers to increasingly employ metabolic and protein engineering to solve a range of ever more complex biosynthetic challenges.

## Identifying and engineering a suitable host organism

A PNP may be selected as a metabolic engineering target for a variety of reasons including medicinal utility, industrial application, or scientific interest. For a given PNP, the first step towards heterologous production is selection of an appropriate host species in which to engineer the pathway. Within a species, use of previously developed strains that overproduce necessary metabolites can greatly accelerate progress. And lastly, within a given strain, preliminary engineering of the host prior to incorporation of heterologous enzymes can facilitate implementation of the non-native pathway in a new context.

### Selecting the host species for a heterologous plant pathway

When selecting a host species for a heterologous pathway, properties such as ease of cloning, ease of culturing, and suitability of the host for the new enzymes and compounds are considered. Organisms with a long history of use in research, and particularly in metabolic engineering, often have well developed techniques for cloning, culturing, and industrial scale-up that make them attractive choices.

A first choice of host for production of PNPs may be plant cells, where plant specific subcellular compartments and protein processing are conserved, a topic recently reviewed elsewhere^7^. Indeed, model plants such as *Nicotiana benthamiana* are useful for transient expression of plant pathway enzymes during preliminary testing and discovery, as enzyme function, necessary cofactors, and substrate pools are likely to be maintained in planta^8,9^. However genetic manipulation of plants, even well-established model plants, remains unwieldy and slow compared to microorganisms and thus a microbial host is often preferable. Microorganisms such as *Escherichia coli* and *Saccharomyces cerevisiae* have a wealth of well-established tools available for genetic manipulation, are easily cultured, have a range of developed platform strains available (see following section, “Selecting a host strain that overproduces PNP precursors”), and are amenable to production scale-up. Other microorganisms are often employed to purposes which evolution has made them especially well-suited: *Streptomyces* is often used for the production of antibiotics originally derived from *Streptomyces* species^10^; *Corynebacterium glutamicum* is widely used for the high-titer production of amino acids^11^; *Yarrowia lipolytica* is frequently employed when using lipids as a substrate^12^. Yet for most applications involving the production of PNPs in a microbial host, *E. coli* or *S. cerevisiae* is employed.

Thus, the most immediate question for a metabolic engineer seeking to produce a compound in a heterologous host is often whether to use *E. coli* or *S. cerevisiae*. Distinct advantages of *S. cerevisiae* are its ease of genomic integration, owing to a high rate of homology directed recombination, and that as a eukaryote yeast contains many organelles found in plants. Some enzymes from PNP biosynthesis pathways, such as cytochrome P450s, are transmembrane proteins and require the presence of an appropriate membrane, such as the endoplasmic reticulum (ER), for proper anchoring and folding. This potential roadblock was demonstrated during the Semi-synthetic Artemisinin Project, a landmark achievement in metabolic engineering in which *S. cerevisiae* was engineered to produce high titers of artemisinic acid, a precursor to the important antimalarial artemisinin. In this project, both *E. coli* and *S. cerevisiae* were considered as potential hosts, and while impressive titers of the intermediate amorphadiene (25 g/L) were achieved in *E. coli*^13^, the subsequent step in the pathway is carried out by P450_AMO_, a plant cytochrome P450. High activity of this enzyme could not be attained in *E. coli* necessitating a switch to production in *S. cerevisiae*^14^. While strategies exist to modify transmembrane proteins for function in the cytosol^15^, using *S. cerevisiae* as a host for pathways containing transmembrane proteins avoids the added labor necessary to modify those enzymes. Furthermore, *S. cerevisiae* contains cellular microcompartments (e.g., mitochondria and peroxisomes) that can be used to mimic the subcellular localization employed in PNP biosynthesis in plants^16^. Conversely, *E. coli* has a doubling time that is 3–4 times shorter than *S. cerevisiae*, is well suited to very high expression of enzymes, and has a different profile of native metabolites available compared to *S. cerevisiae*. For example, the presence of a native pathway for certain isoprenoid compounds was used to engineer *E. coli* strains with 2,400-fold higher production of taxadiene (a precursor to the PNP drug taxol) compared to strains of *S. cerevisiae*^17^ engineered for taxadiene production.

One additional avenue when choosing a host organism for PNP biosynthesis is to utilize multiple organisms in a co-culture with components of a metabolic pathway split between distinct organisms of the same or different species^18–21^. Merits of this approach include reducing burden on the host from the heterologous pathway, the ability to utilize the species most suited to expression of specific enzymes in the pathway, and modularity associated with being able to mix pathways by growing distinct strains together. In one example, benzylisoquinoline alkaloids (BIAs) were synthesized in an *E. coli* and *S. cerevisiae* co-culture system^20^. *E. coli* were engineered for biosynthesis of the branchpoint intermediate (*S*)-reticuline, and *S. cerevisiae* strains were engineered to express membrane-bound P450 enzymes that derivatized (*S*)-reticuline to other PNPs. In another example, high titers of an anthocyanin PNP were achieved by splitting the metabolic burden of the pathway across four *E. coli* strains which were co-cultured^19^. Limitations of co-cultures are pathway specific and include inefficiencies in the transport and/or diffusion of intermediate metabolites between cells in the co-culture and the need to balance growth of multiple hosts in as single culture, which may differ in optimal growth conditions and rates.

### Selecting a host strain that overproduces PNP precursors

Following selection of a host species, engineering the host to increase titers of native metabolites that are biosynthetic precursors to the product of interest can greatly facilitate downstream production of PNP molecules. The core metabolic networks of model organisms are well-characterized and can be used to guide overexpression and knockout modifications for overproduction of central metabolite precursors and to address common challenges (e.g., feedback inhibition or other metabolic regulation). One of the advantages of biosynthesis over chemical synthesis is how readily biosynthetic strains are distributed; once a strain has been engineered to produce a compound, researchers looking to expand on that work in the future need not repeat tedious syntheses of starting material.

Strains of *E. coli* and *S. cerevisiae* that overproduce alkaloids, fatty acids, terpenes, and other valuable compound classes have been engineered (Table 1). Platform strains that overproduce central metabolites or a heterologous secondary metabolite can both be useful: central metabolites, such as geranyl pyrophosphate or amino acids, provide a starting point for the production of potentially thousands of diverse PNP compounds, while secondary metabolites can provide an easy starting point from which to engineer biosynthesis of a specific PNP product. For example, platform strains that produce the key branch point alkaloid *(S)*-reticuline^22,23^ have enabled microbial biosynthesis of a wide range of BIAs produced by *Papaver somniferum* (opium poppy), including morphine^24^ and noscapine^25^. Likewise, strictosidine producing strains^26^ provide a key branch point metabolite for the biosynthesis of monoindole alkaloids (MIAs), which include vincristine, ibogaine, yohimbine, and thousands of others.

**Table 1:** Common platform strains^a^

^a^Examples of engineered strains producing different compounds or compound classes that can be used as platform strains for the production of diverse downstream compounds. Yellow, core metabolite platform; blue, secondary metabolite platform

A platform strain can be useful not only because it produces a valuable starting material, but also because the means of production of said starting material are particularly inexpensive, sustainable, or offer easy handling for the researcher or industrial producer. This is demonstrated by the engineering of an efficient simultaneous saccharification and co-fermentation (SSCF) strain for bioethanol production in *E. coli* which utilizes lignocellulosic biomass^27^, an inexpensive waste product from agriculture and forestry, in place of expensive refined sugars. In another example, an enzyme was designed that allows for assimilation of formate into central metabolism^28^, potentially allowing the biosynthesis of medicines and commodity chemicals from formate, which is expected to be abundantly available from electrochemical reduction of CO_2_. Lastly, researchers generated a strain of *E. coli* that can produce its own biomass from CO_2_ via photosynthesis^29^. Although there is significant interest in utilizing natural photosynthesizing microorganisms (e.g., cyanobacteria) for the production of PNPs^30^, it could be advantageous to engineer the ability to fix CO_2_ in well studied, genetically tractable industrial microorganisms such as *E. coli* and *S. cerevisiae*. While the aforementioned strains have not yet been used for the production of PNPs, the other PNP-producing strains discussed throughout this review could potentially be integrated into these platforms to produce complex PNPs de novo from agricultural waste, formate produced with renewable energy^31^, or directly from atmospheric CO_2_. This principle has been demonstrated through the engineering of *E. coli* to utilize the one-carbon feedstock methanol and ultimately convert it into the flavanoid naringenin^32^. Such strategies could support more sustainable bioprocesses for producing increasingly diverse products, including PNPs, at industrial scale.

### Engineering host metabolism to facilitate PNP biosynthesis

**Fig. 2:**
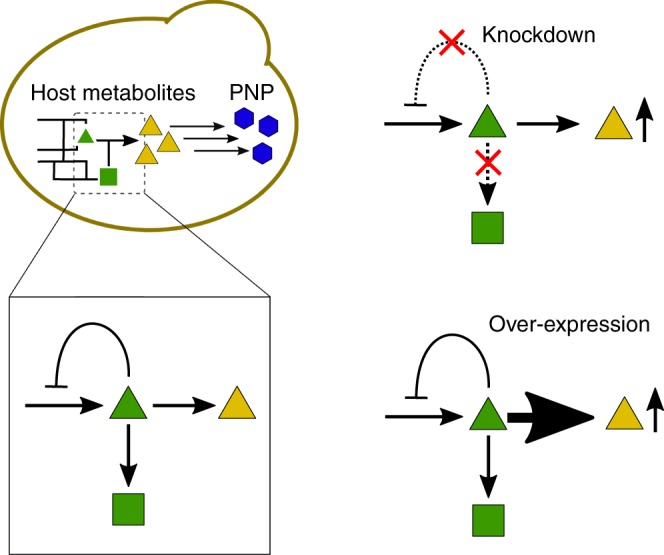
Common host engineering strategies to increase titer of a PNP precursor compound.  Yellow triangles,  core metabolite platform; blue hexagons, secondary metabolite platform

After selection of a host or existing platform strain, the supply of biosynthetic precursors may be enhanced by modifications to the host, such as gene deletions, swapping of endogenous enzymes with more active homologues, or overexpression of endogenous metabolic genes (Fig. 2). A recent *tour de force*^33^ combined all of these techniques to reprogram yeast central metabolism to overproduce acetyl-CoA for isoprenoid and fatty acid biosynthesis - molecules which are a starting point for many PNPs such as the antimalarial artemisinin. A model of the yeast reaction stoichiometries for acetyl-CoA, redox cofactors, and sugar was used to determine a more favorable reaction stoichiometry, which was defined as having a reduced ATP requirement, reduced loss of carbon to side reactions, and improved pathway redox balance. The optimal acetyl-CoA stoichiometry was implemented by augmenting acetyl-CoA biosynthesis with expression of four enzymes involved in acetyl-CoA biosynthesis in other organisms, allowing the yeast to produce 25% more of the isoprenoid farnesene with an equal supply of sugar while requiring less oxygen, an important consideration for oxygen-constrained industrial fermentation environments.

Optimization for tyrosine and *p*-coumaric acid overproduction, from which many PNPs including some alkaloids, polyphenols, and flavonoids are derived, has also been pursued in the context of *E. coli* and *S. cerevisiae*. For example, researchers engineered yeast producing 1.9 g/L of *p*-coumaric acid through a combination of six genetic modifications to yeast native metabolism. These included engineering feedback-resistant enzymes, over-expressing enzymes at bottlenecks, and removing competing side pathways^34^.

Deletion of competing or undesired side pathways in the host is a common strategy to increase precursor titers (Fig. 2). In work on the de novo production of strictosidine, a plant-derived alkaloid, researchers monitored biosynthetic intermediates in their engineered pathway to identify competing side pathway^26^. Finding that geraniol, an intermediate in strictosidine biosynthesis, was metabolized by the yeast through esterification, deletions were made to ATF1 and OYE2 which reduced undesired host interactions and resulted in a 6-fold increase in strictosidine production.

Finally, evolution has emerged as a powerful approach for host optimization, although it has not yet been directly applied to PNP biosynthesis. In the aforementioned work on altering yeast metabolism from alcoholic fermentation to lipogenesis, researchers also employed laboratory evolution methods to improve lipogenic growth on glucose^35^. Deletion of pyruvate decarboxylase genes (PDC1, 5, and 6) involved in alcoholic fermentation resulted in strains unable to grow on glucose as a carbon source. Adaptive laboratory evolution was applied to evolve FFA producing strains lacking ethanol fermentation for growth on glucose by gradually shifting the carbon source from ethanol to glucose over 200 generations. New methods like SCRaMbLE have enabled inducible control of host genetic variation^36^. SCRaMbLE utilizes a synthetic yeast chromosome V with recombination sites introduced in all non-essential genes such that when recombination is induced these genes are shuffled within chromosome V. SCRaMbLE was applied in *S. cerevisiae* and shown to improve host strain background for improved production of violacein, penicillin, and utilization of xylose as a carbon source.

## Strategies for planning and engineering a metabolic pathway

Following selection of a suitable host, a route to the desired PNP can be planned and implemented. A candidate pathway is first outlined through selection of stepwise chemical intermediates leading from host metabolism to the target compound, followed by selection of enzymes to carry out each specified reaction. For certain PNPs, detailed knowledge of the native biosynthetic pathway is available and can be used to outline all intermediates and enzymes in a pathway, facilitating pathway engineering into a heterologous host. However, such detailed knowledge can require years or even decades of dedicated research in planta and is frequently unavailable or incomplete. In such cases, candidate pathway design, enzyme selection, and pathway testing all offer distinct challenges which are discussed in the following sections.

### Computational tools for global pathway design

Literature on a given PNP biosynthetic route can be instrumental to outlining a pathway, although even for well-studied PNPs there are often gaps in our knowledge. One way to overcome the restriction of needing plant biochemical data for each enzymatic step is to use an approach agnostic to the natural product in question. When a reaction path to a chemical entity is unknown, retrosynthetic analysis can be used such that the target molecule is transformed into simpler precursor structures without making assumptions about starting material availability. Resulting precursors are in turn transformed into simpler structures until available starting constituents are reached. By breaking a target molecule into potential precursors, it is then possible to select enzymes which interconvert in the other direction.

Retrosynthetic pathway design deconstructs a PNP one step at a time and utilizes reaction/enzyme pairs from databases such as MetaCyc^37^ to propose biosynthetic routes to the target. Of ten available retrosynthesis-based pathway design tools^38^, only RetroPath^39^ has been experimentally tested. RetroPath takes starting compounds, a target, and reaction rules to generate potential pathways and was experimentally validated on the design of biosynthetic routes to pinocembrin^40^, a flavonoid four enzymatic steps from *E. coli* central metabolism. RetroPath narrowed down a list of nine million in silico pathways to twelve top-ranked candidates, with one providing 24 mg/L pinocembrin after construction and optimization in *E. coli*. Notably, RetroPath and similar tools such as BNICE.ch^24^ only consider the type of reaction occurring when considering enzymatic matches. If the substrate of the desired reaction is very different from that of the known reaction to which it is being compared, ranking the results by some measure of substrate similarly, such as Tanimoto distance, might be advantageous^41^.

Retrosynthesis can also be performed manually, without the aid of automated tools. To characterize the rapidity of heterologous biosynthesis for the production of valuable compounds, a group of researchers recently performed a pressure test to produce 10 molecules of interest in 90 days^42^. The 10 molecules were a mix of PNPs (carvone and vincristine) and non-PNPs; the fungal metabolite epicolactone provides an example of a retrosynthetic approach that could be applied to PNPs to identify potential pathways. The genomic sequence of the native producer of epicolactone was unavailable and so the researchers based their enzymatic retrosynthesis on a previously developed eight-step non-enzymatic chemical synthesis^43^. Enzyme classes were assigned to each reaction manually, guided by literature and pathway databases. Multiple enzymes were identified for each of the eight steps based on reaction type, and to narrow down the candidates, enzyme hits were limited to tropolone-like biosynthetic gene clusters identified from the biosynthetic gene cluster databases MIBiG^44^ and antiSMASH^45^. However, no pathways were experimentally validated within the 90 day time frame.

Nature has developed a limited set of biosynthetic tools; a retrosynthetic scheme might envision chemical transformations which no known enzyme class is able to carry out, and even enzymes which are known to perform the desired chemistry may only do so on a very different substrate. Enzyme evolution to alter substrate scope is still a time-consuming endeavor, and designing enzymes capable of entirely new chemistries has had very limited demonstration thus far^46^. A key question left unanswered for retrosynthetic methods is what strategies can be used for the design of long pathways when some or many steps are non-functional during in vivo testing. This is especially important for the long pathways common in plant secondary metabolism. If automated retrosynthesis tools are to gain more use for PNP biosynthesis, it would be of benefit if not only hypothetical pathways are generated, but also modules containing several enzymatic steps for orthogonal testing, as discussed in the following section “Strategies for the construction of candidate pathways”.

### Computational approaches for enzyme candidate discovery

**Fig. 3:**
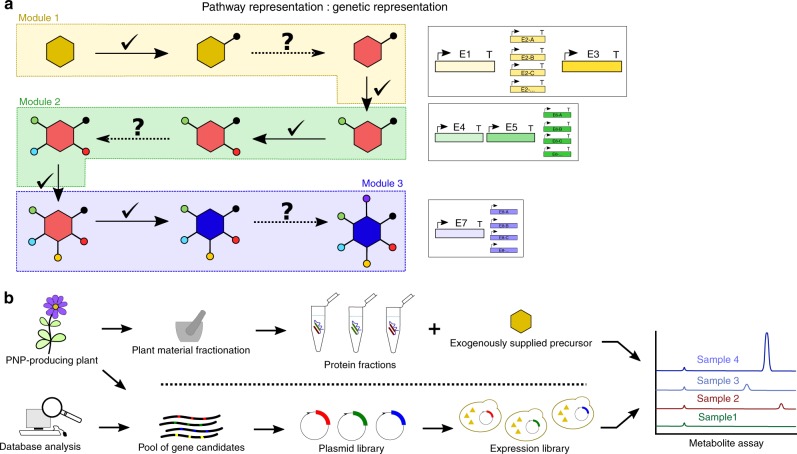
Pathway engineering can be broken down into enzyme module and discovery components. **a** Example of a modular approach to construction of an eight-enzyme pathway; show at the level of pathway and corresponding genetic modules for testing. Note that each module contains 2–3 enzymes, only one of which is unknown (represented by dotted arrow with question mark), thus distributing critical unknown steps amongst the modules to be tested independently. **b** Strategies for identification of an unknown enzyme in a pathway. Starting either with the native PNP-producing plant or a database of genes containing known or putative enzyme activity, a pool of potential genes can be identified and then cloned into a library of plasmids, which are then used to generate a library of host cells expressing the gene candidates. Gene candidates can then be screened for production of the metabolite of interest. Conversely, in the absence of guiding genetic information, the plant material can be fractionated and separated as best as possible into its constituent proteins, to which the precursor for the desired enzymatic transformation is added, and then screened for production of the metabolite of interest. The latter strategy requires additional steps to determine the sequence of the gene encoding the active protein

If the retrosynthetic approach fails to identify functional enzymes for steps in a pathway, enzyme discovery is essential. High-throughput sequencing has enabled efforts to comprehensively profile the genomes and transcriptomes of plant species with important medicinal, industrial, or scientific applications, and these data have fed computational approaches to enzyme discovery, such as plantiSMASH^47^ and the 1KP Project^48^, which leverage genomic information to prioritize biosynthetic gene clusters and enzymes for pathway discovery. Comparison of omics data between species which either produce or lack specific compounds can also help elucidate which enzymes are important for their biosyntheses (Fig. 3).

Demonstrating this approach, two enzymes involved in the biosynthesis of breviscapine flavonoids were discovered entirely via transcriptomic and genomic analysis and subsequently incorporated into an engineered biosynthetic pathway in *S. cerevisiae*^49^. Prior to this work, the enzymes for two steps thought to be catalyzed by a UDP-glycosyltransferase and a P450 were unknown. The researchers identified 83 putative UDP-glycosyltransferases (UDPGTs) from the *Erigeron breviscapus* genome and divided them into 15 gene families. Previous work allowed the researchers to narrow the list from 83 to one likely candidate in the UGT88 family^50^. The function of this lone candidate was validated in vitro and then introduced into a yeast strain producing apigenin, the substrate of UDPGT, resulting in an engineered strain that produced apigenin-7-O-glucuronide. The P450 enzyme was discovered by narrowing down 312 putative P450s in the *E. breviscapus* genome to a list of 134 candidates by comparison with P450s from non-breviscapine producing plant species. Of 134 candidates, 36 were selected and screened for activity using the aforementioned strain producing apigenin-7-O-glucuronide. One P450, CYP706X, resulted in a new peak by HPLC matching the expected product scutellarin, and was subsequently verified via mass spectrometry. This work highlights the ability to identify one enzyme, incorporate it into an engineered strain, and use that new strain to discovery enzymes that perform a subsequent reaction, thus leveraging intermediate strains developed over the course of a project. As DNA synthesis costs continue to drop, one can envision simply synthesizing and testing an entire panel of candidate genes without needing to computationally prioritize the list beforehand.

A similar strategy was employed to discover a key enzyme required for the biosynthesis of morphine in *Papaver somniferum*. During morphinan alkaloid biosynthesis, *(S)*-reticuline is converted to *(R)*-reticuline by an epimerase. In 2015, three different groups reported the discovery of a two-component epimerase, consisting of a reductase and oxidase, using distinct discovery strategies^51–53^. The computational and synthetic biology driven approach taken by one team of researchers^53^ relied on the 1KP Project^48^ and PhytoMetaSyn^25^ databases to search for enzymes similar to a codeinone reductase that had been previously identified. Without accessing plant material, epimerase candidates were synthesized and expressed in an engineered yeast strain producing *(S)*-reticuline, thus affording conversion to *(R)*-reticuline and ultimately enabling de novo biosynthesis of opioids in yeast.

### Experimental approaches for enzyme candidate discovery

Computational approaches to enzyme discovery based on enzyme class, expression, or phylogenetic comparison require a putative enzyme class assignment and/or detailed genomic or transcriptomic data. In the absence of this information, or when the exact nature of the reaction(s) being carried out is unclear, enzyme discovery can be performed with native plant material. This approach is especially important when a reaction involves unique metabolites and/or a catalytic mechanism that is not well represented in enzyme databases.

One powerful approach to discovery is to isolate an unknown enzyme from native plant material via protein fractionation and functional assay^54–56^. The active protein fraction can then be identified using protein-mass spectrometry (protein-MS) followed by transcriptomic or genomic mapping (Fig. 3b). Researchers used this approach to discover an enzyme responsible for the ultimate step in the biosynthesis of thebaine, an opiate alkaloid which is converted to the medicinal opiates codeine and morphine in *P. somniferum*^55^. The conversion of *(7S)*-salutaridinol-7-O-acetate (7SOA) to thebaine can occur spontaneously^57^, but the potential role of an unidentified enzyme in *P. somniferum* had been hypothesized^58^. When latex extract from opium poppy was added to 7SOA an increase in thebaine was measured, indicative of enzymatic activity (thebaine synthase, THS). Because this enzyme catalyzed a reaction previously unknown to biocatalysis, a transcriptomic search based on homologous enzymes was not possible. To isolate the enzyme, protein chromatography was used to enrich fractions with THS activity. Six major proteins were present in the THS active fractions as revealed by protein-MS and comparison with predicted translation products of opium poppy. Each candidate gene was expressed in *E. coli* and tested in vitro, but only one (Bet v1–1) displayed THS activity. Subsequently, the THS variant was expressed in an engineered yeast strain, demonstrating an improved biosynthetic route from fed norlaudanosoline to thebaine.

Another team of researchers used a similar method to discover a UDP-glucose:indoxyl glucosyltransferase (UGIG) for *E. coli* based production of indican^56^, a water soluble indigo precursor with potential application for production of sustainable indigo dye. Purification of UGIG from leaves of *Persicaria tinctoria*, one of the highest yielding plants for indican, led to identification of a UGIG gene. The purified UGIG was analyzed via protein-MS and fragments were matched to transcriptome-predicted sequences. *E. coli* was chosen as a production host based on a prior platform for production of the precursor indoxyl, and UGIG expression in indoxyl producing *E. coli* led to accumulation of indican, validating the role of the discovered enzyme.

Putative enzyme function can also be confirmed using virus-induced gene silencing (VIGS) in planta^54^. This approach was used to identify two unknown enzymes in a seven-step pathway from the MIA tabersonine to the anticancer drug precursor vindoline in *Catharanthus roseus*^59^. Researchers used tissue specific qPCR to determine that only the two terminal steps of the vindoline pathway occur outside of the leaf epidermis. To discover earlier genes in the pathway responsible for a net hydration of the substrate, candidate genes suspected to possess hydratase activity and preferentially expressed in leaf epidermis were queried and two candidate genes were selected. VIGS was used to validate the function of a new oxidase (T3O) and reductase (T3R) enzyme in planta and recombinant enzyme assay showed that product formation was only possible via the coupled action of T3O and T3R. The researchers then used the discovered enzymes to complete a seven-gene pathway in yeast producing vindoline from tabersonine, further validating the functions of T3O and T3R and providing a platform for microbial vindoline production. VIGS has also been used to characterize enzyme activities involved in the biosynthesis of noscapine from *P. somniferum*^60^ and the etoposide aglycone from *Podophyllum hexandrum*^8,60^ among many other examples.

### Strategies for the construction of candidate pathways

Techniques generally used to construct pathways in heterologous hosts are discussed extensively elsewhere^61^; these include discussions of methods for rapid multi-gene integration^62^, gene editing methodologies^63^, and techniques for combinatorial enzyme expression^64^. However, strategies specifically for the organization and testing of long metabolic pathways (defined here as >5 heterologous genes) have not been clearly defined. Pathway planning and enzyme identification, as described in the preceding sections, are useful for selecting enzyme candidates, but transitioning from an outlined pathway to a functional biosynthetic route expressed in a heterologous host is non-trivial^42^. Challenges include the proper expression of candidate enzymes, which may not be functional when expressed in a heterologous host, and the assembly and validation of multi-enzyme pathways when chemical intermediates are not commercially available.

One approach involves breaking a pathway down into biosynthetic modules, where each module’s set of enzymes can be tested and optimized independently in a heterologous host and only combined once validated. Each module ideally begins and concludes with substrates that are commercially available, and steps requiring enzyme discovery are isolated into individual modules, such that a single module is not contingent upon multiple unknown steps (Fig. 3a).

In one example of the utility of clearly defined modules, researchers engineered yeast strains for *(S)*-reticuline production through the use of four genetic modules containing 17 biosynthetic enzymes^53^. The modules focused on overproduction of pathway precursors, cofactor recycling enzymes, production of the intermediate *(S)*-norcoclaurine from native metabolism, and conversion of *(S)*-norcoclaurine to *(S)*-reticuline. This genetic design allowed for independent analysis of each module’s role in the pathway and any limitations. In the same work, a fifth module for thebaine biosynthesis was later designed, possessing additional enzymes that were discovered and engineered independently from the first four modules by feeding *(S)*-reticuline. Ultimately, module five was incorporated into the *(S)*-reticuline producing strain for fully de novo thebaine biosynthesis. A similar modular strategy was employed for the development of other long biosynthetic pathways in heterologous hosts, including for the production of the alkaloids noscapine^65^, sanguinarine^66,67^, strictosidine^26^, and breviscapine flavonoids^49^.

Alongside the pathway specific approach described above, a new frontier for pathway construction is the use of highly automated foundries^68^—collections of wet-lab robotics and software designed to standardize the synthesis, assembly, and testing of DNA parts in microbes. It is unclear if the enzyme discovery components required for some long biosynthetic pathways can be automated, given that discovery methods are frequently tailored to an individual pathway. To date, existing foundry-based approaches have only afforded short pathways (<5 enzymes) or pathways which were already validated^42,69^.

### Enzyme engineering to enable enzyme function in new contexts

When introducing enzymes into a heterologous host, an enzyme may function suboptimally or not at all for reasons that include the new host context (improper folding, post-translational modifications, mislocalization, missing cofactors) or the new chemical context (suboptimal pH, non-natural substrate present, product feedback inhibition). Sub-optimal function of a heterologous enzyme may result in bottlenecks of carbon flux from central metabolism into the PNP pathway. Many of these modes of failure can be alleviated through enzyme engineering.

In plants, localization can cluster pathway enzymes, separate reaction intermediates in the pathway, and provide specific pH or substrate conditions. Localization can be a powerful tool when expressing plant enzymes in microbial contexts for the same reasons. In engineering a heterologous yeast strain for the production of morphine and its semi-synthetic derivatives, researchers observed substantial accumulation of the undesired side product neomorphine^70^. Neomorphine accumulation results from activity of codeinone reductase (COR) on the direct product of T6ODM, neopinone, prior to a spontaneous double bond shift. ER localization tags were fused to COR, thereby sequestering it in the ER and allowing more time for cytosolic neopinone to spontaneously rearrange to codeinone before interacting with COR. The localization strategy ultimately increased morphine titers by sevenfold while decreasing production of the undesired intermediate neomorphine by fourfold. Engineering spatial organization of enzymes can also be accomplished through the use of synthetic scaffolds, which can increase product titers through enzyme clustering or increased local substrate concentration. In one example, researchers constructed a three enzyme pathway in *E. coli* from acetyl-CoA to the intermediate mevalonate, a precursor to the important PNP artemisinin^71^. Mevalonate titers were improved by 77-fold to 5 mM by using SHL/SH3 association domains to cluster the three enzymes in the pathway. RNA-based scaffolds have also been applied to cluster enzymes on RNA-scaffolds using RNA-binding domains fused to enzymes^72^.

Another common problem faced when expressing plant enzymes in a new context is host misprocessing of post-translational modifications or signal peptides. In the course of engineering the biosynthesis of opioids in yeast, researchers encountered low activity in the enzyme salutaridine synthetase (SalSyn)^53^. Western blotting indicated yeast-expressed SalSyn was present as three distinct molecular weights resulting from improper N-linked-glycosylation, indicative of improper localization to the lumen of the ER instead of the ER outer membrane. Protein engineering corrected the improper N-terminal sorting of SalSyn, allowing it to localize to the ER outer membrane and preventing N-linked glycosylation. The engineered enzyme improved conversion of (*R*)-reticuline to salutaridine by sixfold. In another example, researchers engineered brewer’s yeast for biosynthesis of aromatic monoterpene molecules (linalool and geraniol) native to the hop plant and important components to the flavor of beer^73^. In plants, monoterpene biosynthesis occurs in chloroplasts and plant monoterpene synthases typically contain N-terminal plastid targeting sequences (PTSs) of 20–80 amino acids which are cleaved to yield mature protein. In the absence of PTS cleavage, enzyme function is decreased. The researchers tested truncated linalool synthases using bioinformatic and structural information to predict the PTS sites for removal. In one instance, truncation of the PTS motif resulted in a 15-fold improvement in linalool titers. In the same work, additional enzyme engineering was carried out on HMG-CoA reductase, a key rate-limiting step in the pathway for monoterpene biosynthesis. HMG-CoA reductase is controlled by an allosteric domain which responds to product accumulation by inhibiting enzyme function. The researchers truncated the yeast HMGR protein removing an inhibitory domain, thereby increasing flux towards end products.

In addition to the rational modifications discussed, additional enzyme engineering may be required for increasing yield or enabling an enzyme to act on a non-native substrate. The latter might be encountered for enzymes candidates selected through RetroPath or similar computational approaches. Techniques for engineering enzymes with higher activity or for promiscuity toward non-native substrates is a subject covered in the next section and detailed in other recent reviews of enzyme engineering^74^.

## Leveraging engineered strains to make novel PNP derivatives

**Fig. 4:**
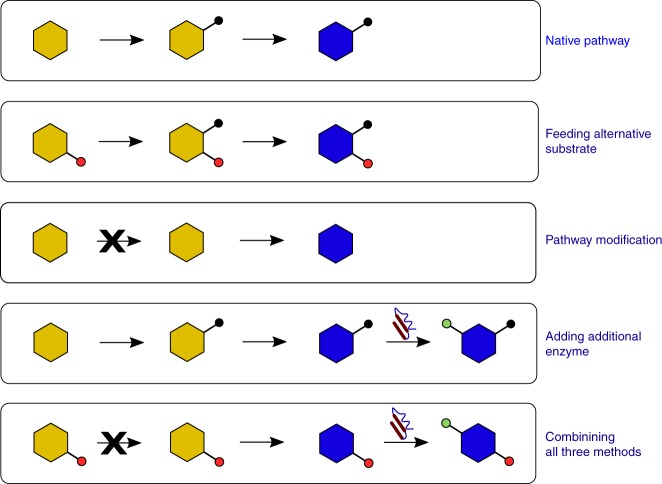
Potential means of producing novel metabolites once a heterologous pathway to a natural PNP is produced in a microbial host. An example heterologous PNP biosynthetic pathway (top row) can be leveraged to produce novel products in a number of ways: (1) feeding unnatural substrates which are then functionalized by downstream enzymes (second row), (2) removing pathway enzymes to exclude biosynthetic steps (third row), or (3) adding additional enzymes to functionalize intermediates or the final product (fourth row). These three strategies can then be combined in order to afford a wealth of additional products (fifth row)

The previous sections have discussed engineering microorganisms to make a desired PNP. Once established in a genetically tractable microbial host, heterologous biosynthetic pathways are an invaluable resource for the synthesis of new-to-nature molecules. While PNPs often possess useful biological activities and accordingly are frequently employed directly as drugs, a higher percentage of drugs are derivatives of PNPs^75^. Derivatization can enhance biological properties of a compound, but PNPs are often synthetically complex, precluding practical syntheses of novel derivatives. With an established heterologous biosynthetic pathway, one can readily replace, add, or remove enzymes or feed in alternative starting materials to synthesize functionalized derivatives. The following sections discuss strategies by which an established PNP-producing engineered microorganism can be leveraged to produce new-to-nature molecules. Each strategy is potentially complementary and in theory can afford a wealth of novel chemical entities from a single starting heterologous pathway (Fig. 4).

### Novel PNP derivatives via unnatural substrate feeding

In addition to the ease of genetic modification of industrial microorganisms, including *E. coli* and *S. cerevisiae*, liquid cultures are easily fed exogenous substrates for incorporation into engineered biosynthetic routes. By extension, derivatives of pathway intermediates can be fed to access derivatives of downstream products. For example, feeding the unnatural intermediate norlaudanosoline to yeast and expressing three methyltransferases resulted in the native intermediate *(S)*-reticuline, demonstrating the flexibility of some enzymes to accept derivatives of their native substrates^76^.

However, substrates which differ more radically from the native substrate are less likely to be accepted at high efficiency, though enzymes differ greatly in their promiscuities. Promiscuity can be assayed in vitro, as was done for the BIA biosynthetic enzyme norcoclaurine synthase^77^ and the enzymes for *(S)*-reticuline epimerization to *(R)*-reticuline^51^, or in vivo, as was done for the MIA biosynthetic enzyme strictosidine synthase^78^. In the former cases, the enzyme in question was purified and reacted with derivatives of the native substrate in vitro, thus elucidating which substrates are likely to be accepted in vivo. A cell-free system was developed to assay the ability of prenyltransferases to produce PNPs and novel derivatives and was demonstrated with cannabinoids from *Cannabis sativa*; by feeding in divarinic acid in place of olivetolic acid, cannabinoids that are typically minor products in planta were produced in high titers and prenyltransferase mutants were quickly assayed for substrate selectivity^79^. Promiscuity is also readily probed in vivo directly in the pathway context. Novel isoflavonoids have been produced through feeding of flavanones to engineered yeast^80^, while novel flavonoids and stilbenes were similarly generated from carboxylic acids fed to engineered *E. coli*^75^. In their work on the de novo biosynthesis of noscapine, researchers showed that several halogenated derivatives of the early intermediate tyrosine were accepted by seven downstream enzymes in the pathway, affording halogenated derivatives of pathway intermediates up to (*S*)-reticuline^5^. The substitution of a hydrogen atom for a halogen is relatively sterically conservative, but is a ubiquitous modification in medicinal chemistry, with nearly a quarter of all pharmaceuticals containing at least one halogen^81^. However, the titer of the halogenated reticuline derivatives was either too low for halogenated derivatives of further downstream intermediates to be observed, or the subsequent enzyme, berberine bridge enzyme, possesses too narrow a substrate scope. When derivatization occurs at the terminus of a pathway, enzyme promiscuity may not be required. For example, researchers produced novel betalain pigments in yeast by feeding diverse amine scaffolds^82^. A yeast strain was engineered for betalamic acid production, which then spontaneously condensed with the fed primary and secondary amines resulting in new-to-nature pigments.

### Novel PNP derivatives via combinatorial biosynthesis

Once a heterologous biosynthetic pathway is established, the enzymes in that pathway and/or analogues of those enzymes can be employed in different combinations to afford distinct products. In this way, no unnatural substrates or novel enzymes need be introduced; all of the necessary tools to make new products are present from the initial engineering effort. For example, in biosynthesis of noscapine from canadine in *S. cerevisiae*, expressing one cytochrome P450, CYP82Y1, in the absence of the preceding enzyme, an N-methyltransferase, afforded 1-hydroxycanadine in place of the usual product, 1-hydroxy-N-methylcanadine^65^. Similarly, swapping CYP82Y1 with CYP82X2, which is downstream in the native biosynthetic pathway, resulted in the production of N-methylophiocarpine, an isomer of the native product, 1-hydroxy-N-methylcanadine. Neither of these two products had previously been identified in the native plant host, *P. somniferum*^65^. The combinatorial space around terpene biosynthetic pathways has been similarly probed using transient expression in *Nicotiana benthamiana* to generate novel sesquiterpenoids derived from the parthenolide biosynthetic pathway from feverfew (*Tanacetum parthenium*)^83^. This space can be further expanded through the introduction of analogues of the native pathway enzymes. Researchers reconstituted the rebeccamycin biosynthetic pathway from the soil bacterium *Lechevalieria aerocolonigenes* in *Streptomyces albus*, which natively contains RebH, a tryptophan 7-halogenase^84^. By exchanging RebH with pyrH and thal, a tryptophan 5-halogenase and a tryptophan 6-halogenase, respectively, and expressing the other pathway genes in different combinations, a total of 32 different compounds were produced. A similar strategy could be applied to PNPs using *S. cerevisiae* or *E. coli* as a host to rapidly explore structure space around the native PNP.

### Novel PNP derivatives via novel enzyme incorporation

The chemical space accessed by heterologous pathways rebuilt from plants can be further expanded by addition of new enzymes to the pathway. In this way, a natural product can be directly transformed via halogenation, hydroxylation, methylation, prenylation, or any other chemistry available via an enzyme that will accept the natural product as a substrate. Since natural products are often large and complex, an enzyme which natively performs the desired transformation at high efficiency may not be available. However, protein engineering can be used to expand the substrate scope of existing enzymes to accept larger substrates, as has been demonstrated for cytochromes P450^85^, halogenases^86^, and aminotransferases^87^, to name only a few classes. Through similar efforts, type III polyketide synthases have been engineered to produce novel and often larger products by accepting novel substrates and performing additional chain elongation steps prior to cyclization^88–91^. Given the large effort traditionally required for protein engineering and metabolic engineering, though, relatively few examples in which the two techniques are successfully combined have been reported. In light of advances that have accelerated both and furnished a wealth of engineered enzymes and pathways with which to work, we can expect to see the two utilized in concert increasingly in the future. For example, a recent perspective discussed valuable drugs that could be produced by leveraging opioid biosynthetic *S. cerevisiae* strains with engineered enzymes and subsequent semi-synthesis^92^; these include the pharmaceuticals cisatracurium, levorphanol, and butorphanol.

To date, metabolic pathways have been more frequently modified with natural enzymes. For example, in work on the combinatorial biosynthesis of the bacterial metabolite rebeccamycin in *E. coli*^42^, researchers identified 21 additional genes known to modify the bisindole core and predicted that combinatorial expression of those enzymes could access to a total of 540 bisindole derivatives, 98% of which have not yet been reported in PubChem. Early concrete demonstrations of addition of derivatizing enzymes to metabolic pathways mostly utilize pathways in their native host, rather than in heterologous hosts. For example, researchers added a halogenase to the marine bacterium *Streptomyces coeruleorubidus*, which natively produces pacidamycin, in order to produce the new-to-nature derivative chloropacidamycin^93^. The researchers used the introduced chlorine as a synthetic handle for cross-coupling reactions to make a range of novel products. While the two preceding examples are of bacterial natural products, the strategies could readily be applied to PNP pathways expressed in microbial hosts. In a notable achievement, researchers reported the integration of two halogenases into the medicinal plant *Catharanthus roseus* and observed that chlorinated catharanthine alkaloids were produced^94^. Both of the preceding examples rely on the halogenation of tryptophan, an early intermediate in the pathway; in *C. roseus*, 7-chlorotryptophan accumulation was observed in the plant and suspected to adversely affect the growth rate. To alleviate this, researchers engineered RebH to act on tryptamine, the immediate downstream metabolite in the biosynthetic pathway, rather than on tryptophan^95^; integration of this engineered RebH variant showed no accumulation of 7-chlorotryptophan. More recently, researchers engineered *E. coli* to produce resveratrol, a stilbenoid produced by several plants, and then added the halogenase Rdc2 to produce 2-chlororesveratrol^96^. This work utilized a heterologous pathway in an industrial microorganism with an additional enzyme introduced to produce a new-to-nature natural product derivative, albeit with a relatively simple chemical structure. Given the dramatically more complex PNPs that have been biosynthesized heterologously in recent years, future demonstrations of non-native enzyme incorporation will furnish increasingly synthetically complex novel products.

## Conclusion and future directions

The past 20 years of PNP metabolic engineering have seen increasingly sophisticated pathway engineering, with engineered pathways composed of two to seven enzymes in the early 2000s progressing to pathways containing 20 or more enzymes at present. The more enzymatic steps in a heterologous pathway, the more formidable the challenge for construction of the pathway, discovery of the requisite enzymatic components, and overcoming interdependencies introduced between the many enzymatic steps. Taken at face value, this should mean that long, complex PNP biosynthetic pathways take significantly longer to engineer than simple pathways. However, recent years have seen the rapid implementation of long pathways as a result of advances in DNA synthesis, sequencing, and genome engineering. These enabling technologies, along with the emergence of PNP platform strains, have allowed the discovery and engineering of increasingly long PNP pathways.

Following these trends, the field of metabolic engineering leverages sequencing and synthesis to more rapidly discover pathways and enzymes and engineer those pathways into metabolic hosts. Decreasing costs of high-throughput sequencing continue to allow comprehensive profiling of plant genomes and transcriptomes, providing plentiful putative enzyme targets that can be mined via comparison with existing databases of enzymes of known function. Inexpensive DNA synthesis enables wholesale synthesis of dozens of predicted enzymes for any given step in a pathway. This approach has the advantage that hundreds of hypothesized enzymes can be tested for a given step as opposed to testing individual enzymatic hypotheses in planta. Platform strains producing important metabolites for a given specialized PNP pathway will be used to screen single enzymes and combinations of predicted enzymes to reconstitute partial metabolic pathways. Importantly, once a platform strain for a given intermediate is made, the discovery and assembly of downstream pathways are greatly facilitated.

Throughout this review, we have highlighted instances in which protein engineering was employed to solve challenges (e.g., low activity, product inhibition, and poor functioning in new host conditions) encountered during the reconstruction of metabolic pathways in heterologous hosts. As the design-build-test cycles associated with both metabolic engineering and protein engineering have been greatly accelerated in recent years, we expect to see protein engineering employed more frequently during the construction of heterologous metabolic pathways. Although these cycles have accelerated, engineering enzymes for altered small-molecule production still relies on screening, typically via LC or GC, rather than selection, resulting in a disproportionate amount of project time required for sample analysis, even for limited library sizes. One exciting means to engineer selections for small molecules is through the use of genetically-encoded biosensors linking concentration of a compound of interest to an output such as fluorescence-protein expression or cell fitness. Protein and RNA biosensors can be engineered to recognize a range of small molecules and control genetic output accordingly^97–99^, and early examples have demonstrated their application in enzyme evolution^23,100,101^. As methods for developing biosensors improve we anticipate that they will be increasingly employed in enzyme engineering to explore wider ranges of sequence space more rapidly than traditional screening methods allow. Furthermore, we expect protein engineering to be used to solve a wider range of problems in the future—not only to adapt existing enzymes to their new conditions, but also to develop completely novel activities which fill in gaps in biosynthetic pathways or expand pathways in new directions for the production of novel PNP derivatives. This will allow existing and future heterologous biosynthetic pathways to be leveraged for the production of innumerable valuable and novel chemical entities. As metabolic engineering accelerates towards increasingly complex and tailored pathways in the coming years, we expect protein engineering to become an increasingly dominant force for the production of known and novel molecules on both the research and industrial scales.
